# Author Correction: Inversions of landslide strength as a proxy for subsurface weathering

**DOI:** 10.1038/s41467-023-37014-w

**Published:** 2023-03-17

**Authors:** Stefano Alberti, Ben Leshchinsky, Josh Roering, Jonathan Perkins, Michael J. Olsen

**Affiliations:** 1 Department of Forest Engineering, Resources and Management, Oregon State University, Corvallis, OR 97331 USA; 2 Department of Earth Sciences, University of Oregon, Eugene, OR 97403 USA; 3 U.S. Geological Survey, Moffett Field, Mountain View, CA 94035 USA; 4 School of Civil and Construction Engineering, Oregon State University, Corvallis, OR 97331 USA

**Keywords:** Natural hazards, Geomorphology

Correction to: *Nature Communications* 10.1038/s41467-022-33798-5, published online 13 October 2022

The original version of this Article contained an error in the Acknowledgements, which incorrectly read ‘B.L. and M.J.O. acknowledge support from the Oregon Department of Transportation from SPR807 and SPR 808 and National Science Foundation Grant ECI1929304.’ The correct version states ‘CMMI-2050047’ in place of ‘ECI1929304’. This has been corrected in both the PDF and HTML versions of the Article.

