# Supplementary material for: Inversions of landslide strength as a proxy for subsurface weathering
Source: Nat Commun. 2022 Oct 13;13:6049. doi: 10.1038/s41467-022-33798-5 (PMC9561700; doi:10.1038/s41467-022-33798-5)
Supplement: Supplementary file 1 — Supplementary Information [file 41467_2022_33798_MOESM1_ESM.pdf]

Supplementary Information for:

Inversions of Landslide Strength as a Proxy for Subsurface Weathering

Stefano Alberti[1], Ben Leshchinsky[1]*, Josh Roering[2], Jonathan Perkins[3] & Michael J. Olsen[4]

[1]Department of Forest Engineering, Resources and Management, Oregon State University, Corvallis, Oregon 97331, USA

[2]Department of Earth Sciences, University of Oregon, Eugene, Oregon 97403, USA

[3]U.S. Geological Survey, Moffett Field, California 94035, USA

[4]School of Civil and Construction Engineering, Oregon State University, Corvallis, Oregon 97331, USA

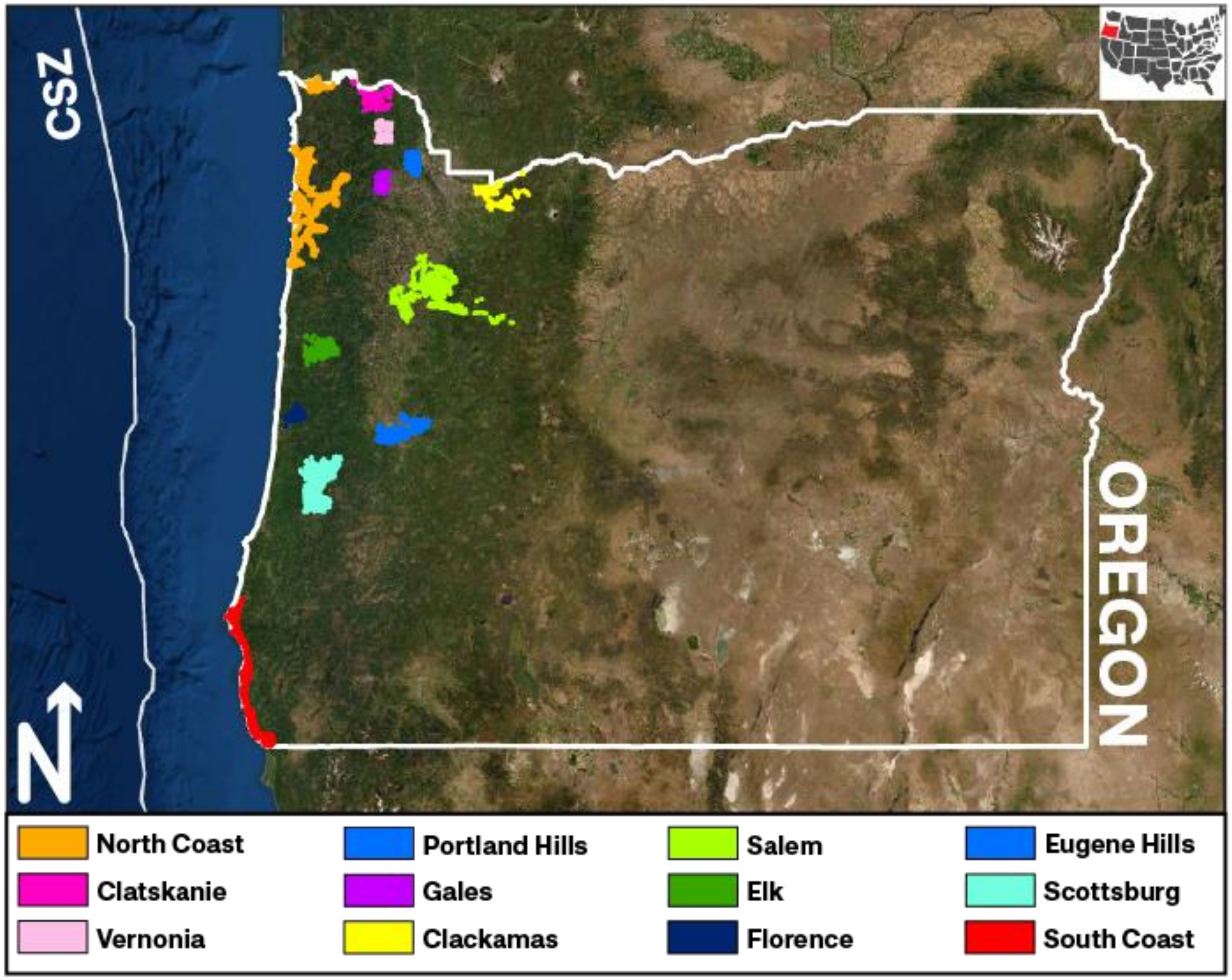

Figure SI.1. Map of landslide inventories. Manually mapped landslides (n=7,330) from the study areas shown with different colors satellite image of Oregon, USA (basemap sources: *Esri, DigitalGlobe, GeoEye, i-cubed, USDA FSA, USGS, AEX, Getmapping, Aerogrid, IGN, IGP, swisstopo, and the GIS User Community*). In the Pacific Northwest of the United States, magnitude 8 (M8) to M9 earthquakes occur every 300 to 500 years along the Cascadia Subduction Zone (CSZ), a 1,100-km long megathrust fault that accommodates subduction of the Juan de Fuca Plate beneath the North American plate (Madin et al 2013). Landslide data are from SLIDO (Statewide Landslide Information Database for Oregon by the Oregon Department of Geology and Mineral Industries, v.4.2 released on 10/30/2020m, Franczyk et al. 2019).

Table SI.1. Median morphological values of reconstructed landslide source areas and deposits used in analysis, sorted by classification (Franczyk et al. 2019).

LANDSLIDE SOURCE				
	Mean Thickness (m)	Mean Surface Inclination (°)	Area (m^2)	Volume (m^3)
Bedrock Translational Landslides	2.54	24.3	11634	29734
Bedrock Rotational Landslides	2.59	20.3	15129	39456
Soil/Debris Translational Landslides	0.92	21.8	2019	1855
Soil/Debris Rotational Landslides	1.17	21.2	2413	2355
Earth Flows	1.53	19.8	5615	8641
Rock Flows	1.82	21.2	10555	19406
Complex Landslide Movement	3.77	21.2	24786	92628
All Landslides	2.57	21.3	13920	35423

LANDSLIDE DEPOSIT				
	Mean Thickness (m)	Mean Surface Inclination (°)	Area (m^2)	Volume (m^3)
Bedrock Translational Landslides	3.16	22.0	12803	37298
Bedrock Rotational Landslides	3.33	18.2	16476	54456
Soil/Debris Translational Landslides	1.06	19.7	2003	2355
Soil/Debris Rotational Landslides	1.46	18.8	2627	3114
Earth Flows	1.78	17.6	6020	9474
Rock Flows	2.23	19.1	11849	27823
Complex Landslide Movement	4.82	19.4	29503	141922
All Landslides	3.15	19.2	15748	48145

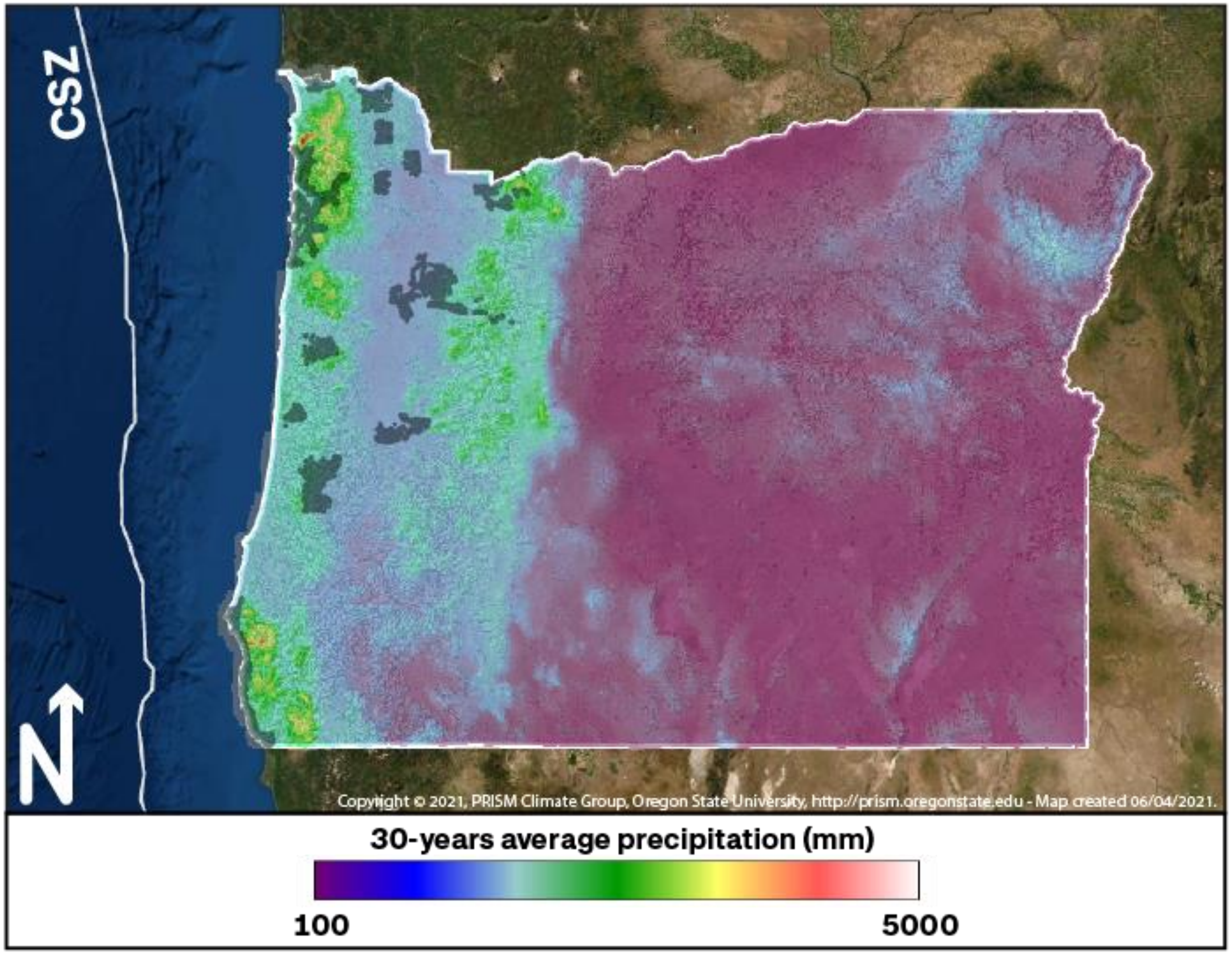

Figure SI.2. Map of mean annual precipitation for the past thirty years. Landslide inventories are shaded in gray (basemap sources: *Esri*, *DigitalGlobe*, *GeoEye*, *i-cubed*, *USDA FSA*, *USGS*, *AEX*, *Getmapping*, *Aerogrid*, *IGN*, *IGP*, *swisstopo, and the GIS User Community*). Precipitation data comes from the PRISM Climate Group, Oregon State University, http://prism.oregonstate.edu, created 6 June 2021.

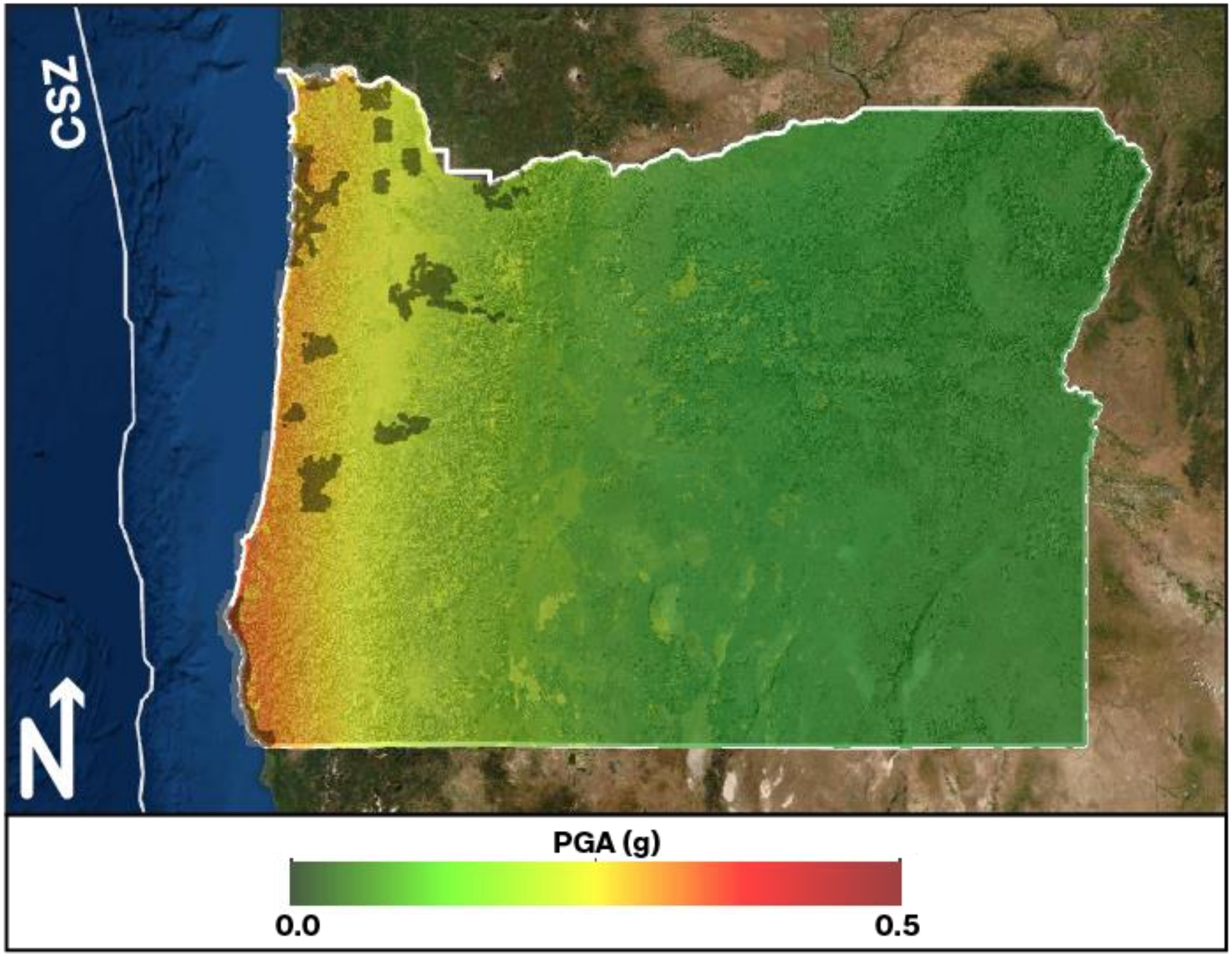

Figure SI.3. Map of modeled peak ground acceleration (PGA) for a magnitude 9.0 CSZ rupture event (basemap sources: *Esri*, *DigitalGlobe*, *GeoEye*, *i-cubed*, *USDA FSA, USGS, AEX, Getmapping, Aerogrid, IGN, IGP, swisstopo, and the GIS User Community*). Landslide inventories are shown in shades of gray. This map uses bedrock PGA values from the model developed by the U.S. Geological Survey for a specific scenario of Cascadia subduction earthquake ground shaking in the Pacific Northwest (Madin et al. 2013).

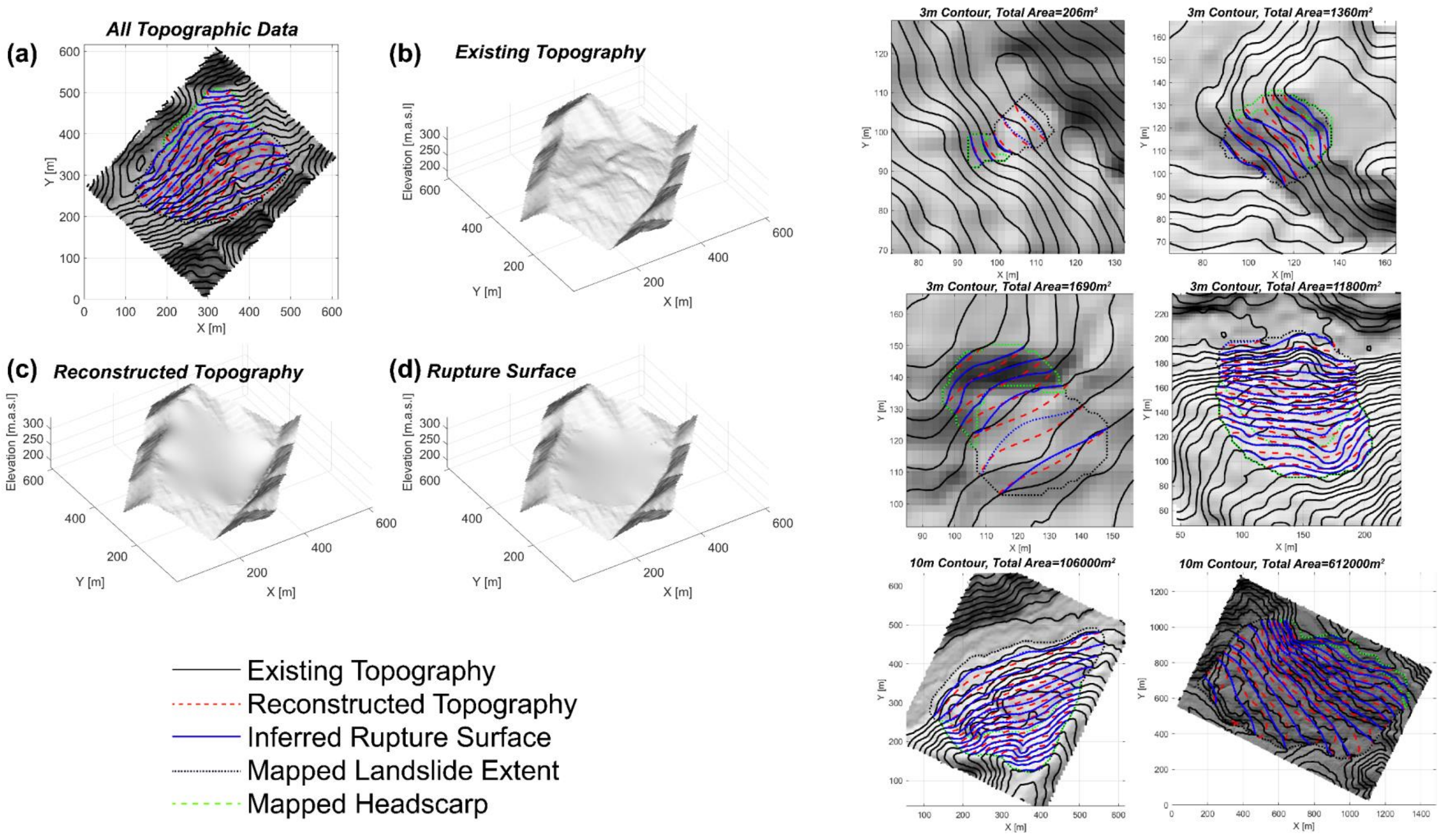

Figure SI.4. (a) Example of landslide rupture surface and topographic reconstruction technique. This specific example is a landslide with a source (scar) area and volume of approximately 4.81×10^4 m^2 and 4.79×10^5 m^3, respectively. (b) Contours of existing topography (solid black contours), (c) pre-landslide reconstructed topography (red dashed contours), (d) inferred rupture surface geometry (blue dashed contours), and the inventoried landslide extent (thin, black dashed line). Contour elevations are presented in meters above sea level (m.a.s.l.). Existing surface topography with landslide extent marked by a red, dashed line. Reconstructed surface topography using curvature-preserving inpainting technique with landslide extent marked by red, dashed line. Mapped rupture surface topography determined from using a thin-plate spline technique with landslide extent marked by red, dashed line and the scarp mapped by the green dashed line. **(Right Panel)** Contours for a series of different landslide sizes reflecting existing topography, the inferred rupture surface and reconstructed topography.

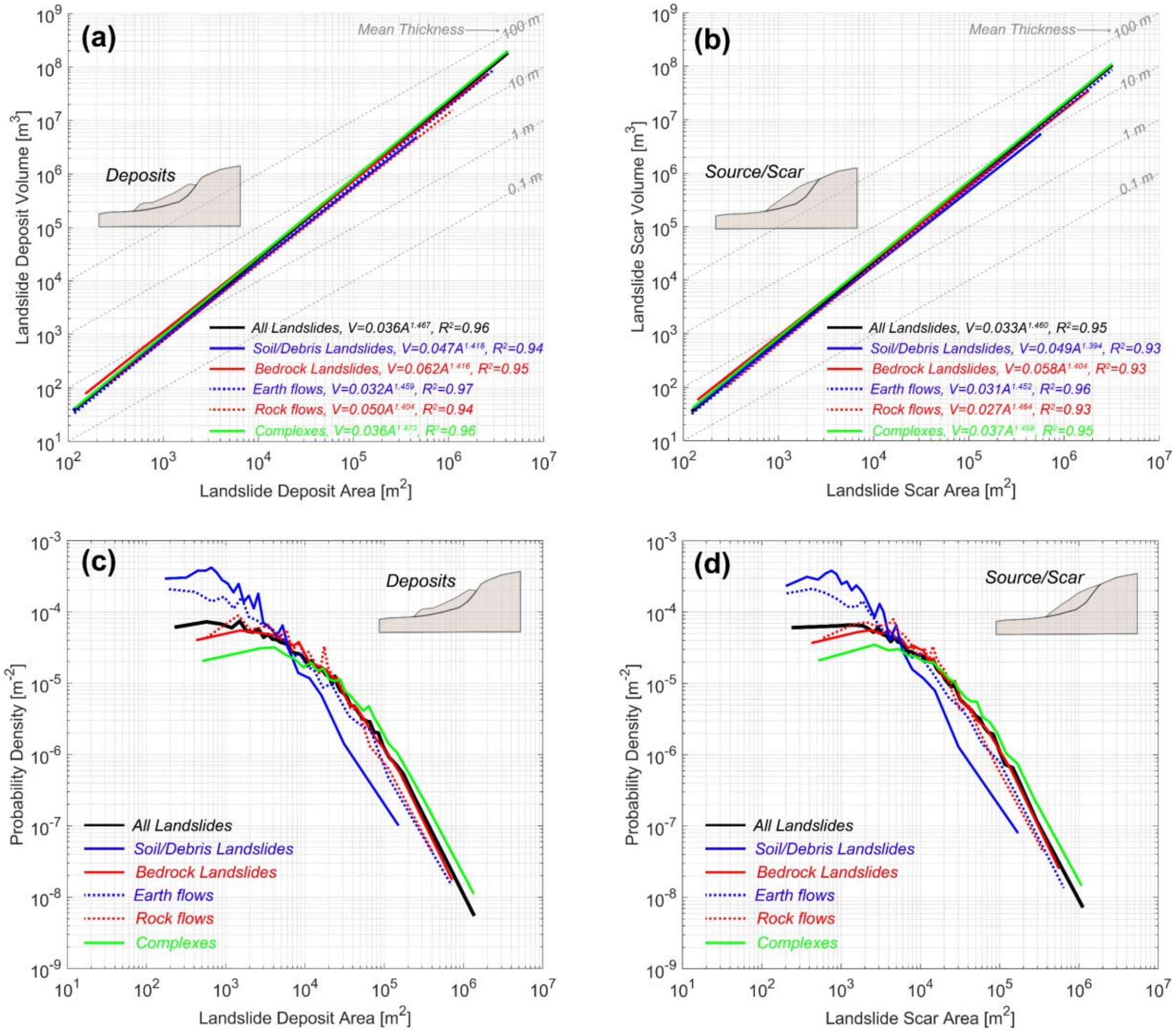

Figure SI.5. (a) Area-volume relationships based on landslide deposits. **(b)** Area-volume relationships for landslide source areas. **(c)** Magnitude-frequency relationships of landslide area based on deposits for all landslides (gray line), soil/debris landslides (blue line), and bedrock landslides (red line). **(d)** Magnitude-frequency relationships of landslide area based on reconstructed scar/source topography for all landslides (gray line), soil/debris landslides (blue line), and bedrock landslides (red line). The rollover behavior reflects significant censoring of smaller landslides, which are potentially more comprised of soil.

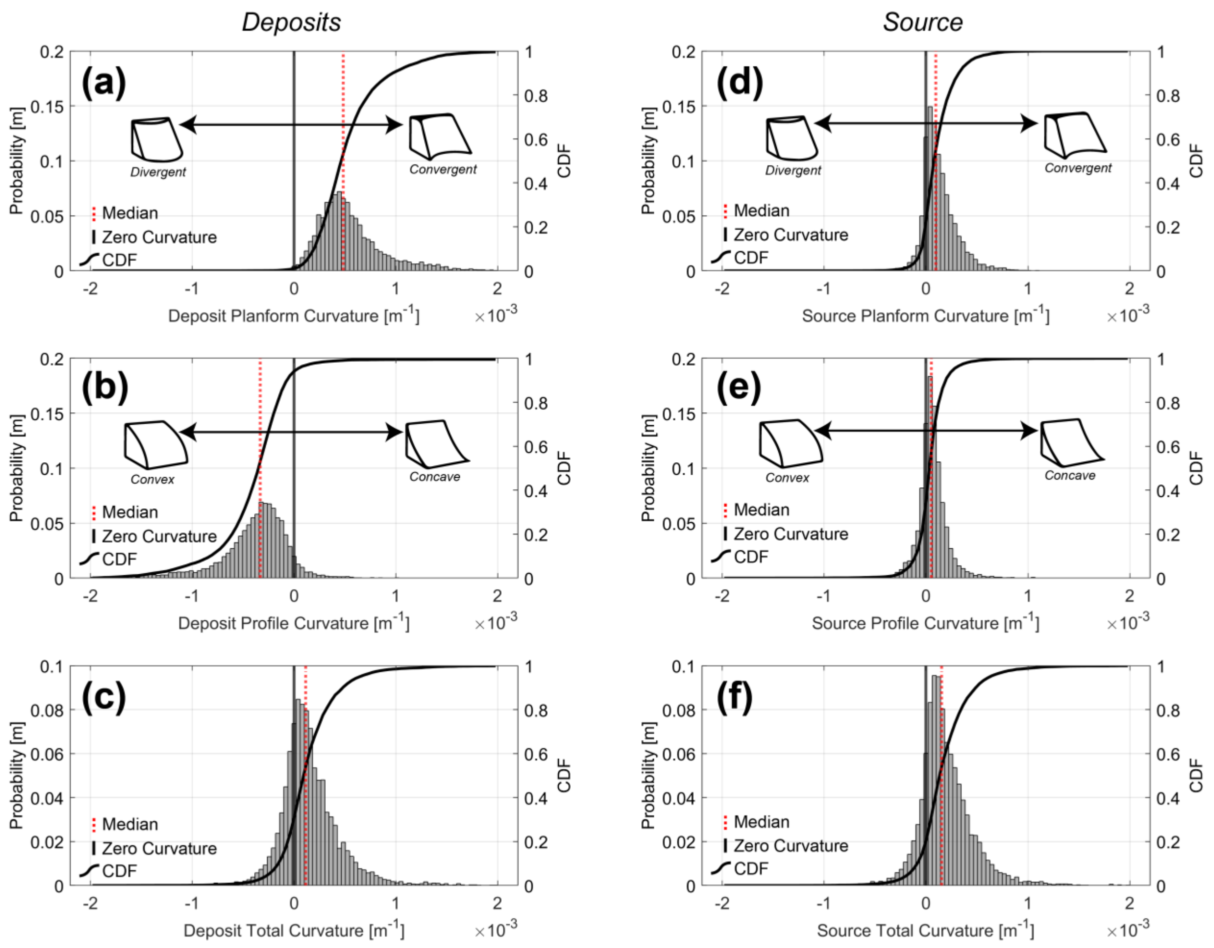

Figure SI.6. Distributions of **(a)** planform, **(b)** profile, and **(c)** total surface curvature for landslide deposits. Distributions of **(d)** planform, **(e)** profile, and **(f)** total surface curvature for landslide source. The median curvature is shown with a red, dashed line. A cumulative distribution function (CDF) is shown to reflect the distribution of curvatures.

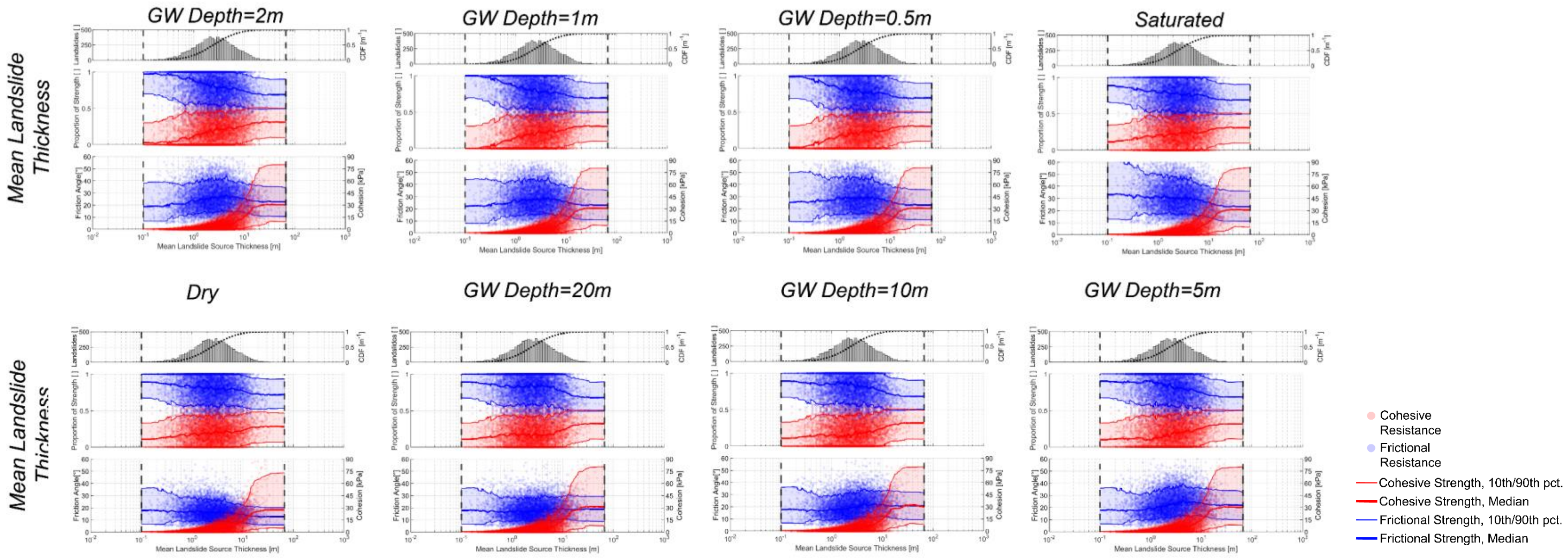

Figure SI.7. Relationships between landslide area versus strength and mean landslide thickness versus strength for a suite of groundwater conditions. We consider dry conditions, groundwater depths of 20m, 10m, 5m, 2m, 1m, 0.5m, and full saturation (0 m depth to groundwater). The exchange in proportional frictional and cohesive strength is largely insensitive to the groundwater conditions shown, although a modest increase in cohesive resistance is observed with decreasing groundwater depth. No evident exchange in proportional strength is observed for the range of landslide areas shown, but the trends are more apparent when comparing proportional strength to mean landslide thickness. Magnitudes of shear strength are sensitive to groundwater conditions. Generally, a modest decrease in friction angle and significant increase in cohesion is observed with increasing mean landslide thickness, but is diminished for discrete groundwater depths. A decrease in friction angle and modest gain in cohesion is shown with increasing landslide area for all groundwater conditions.

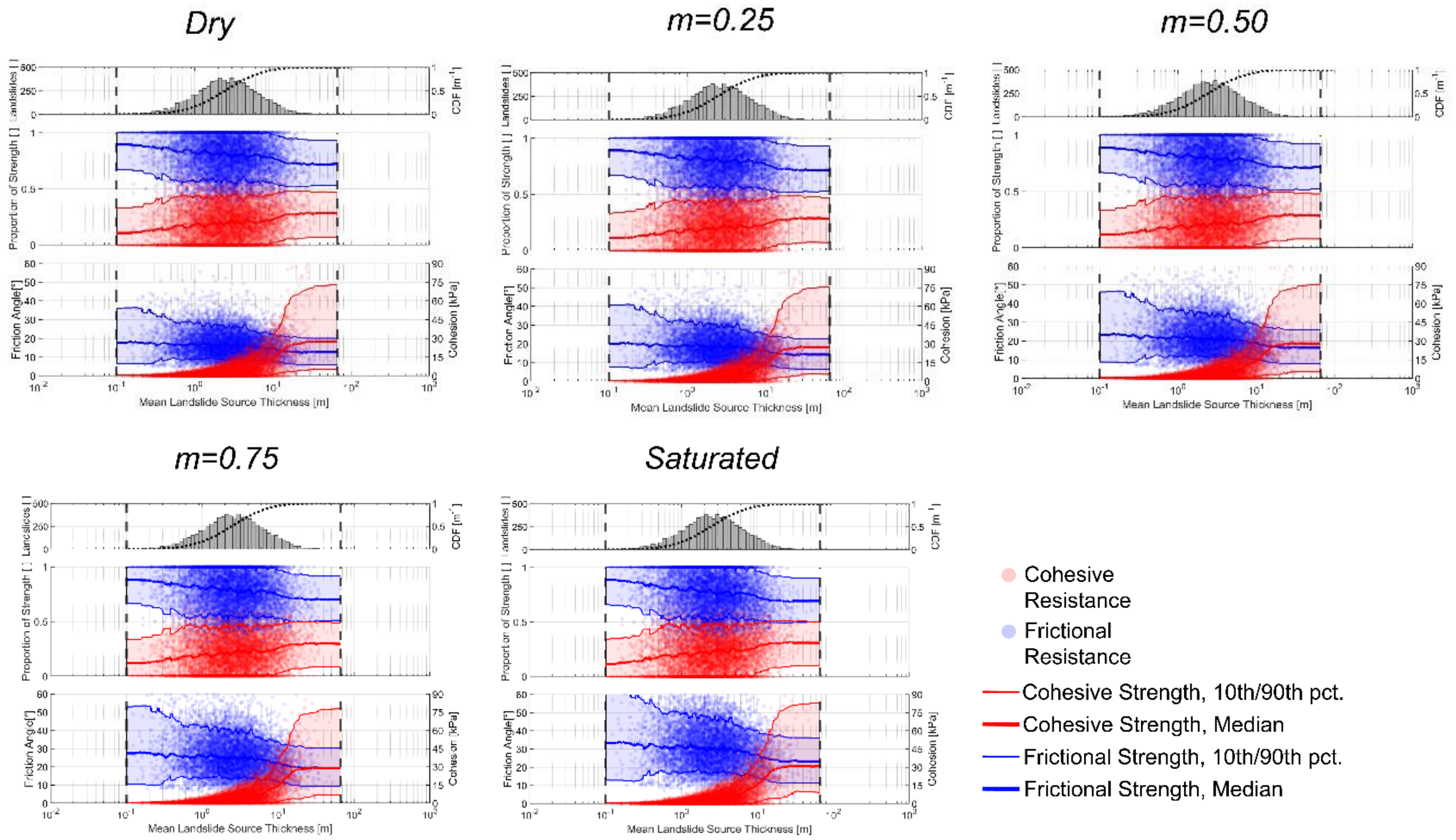

Figure SI.8. Relationships between mean landslide thickness versus strength for a suite of saturation ratios (*m*). We consider dry conditions, pore pressure ratios of 0.25, 0.5, 0.75, and full saturation. Comparatively, the exchange in proportional frictional and cohesive strength is largely insensitive to the pore pressure ratios shown, although a modest increase in cohesive resistance is observed with increasing r_u. Magnitudes of shear strength are sensitive to *m*. Generally, a modest decrease in friction angle and significant increase in cohesion is observed with increasing mean landslide thickness.

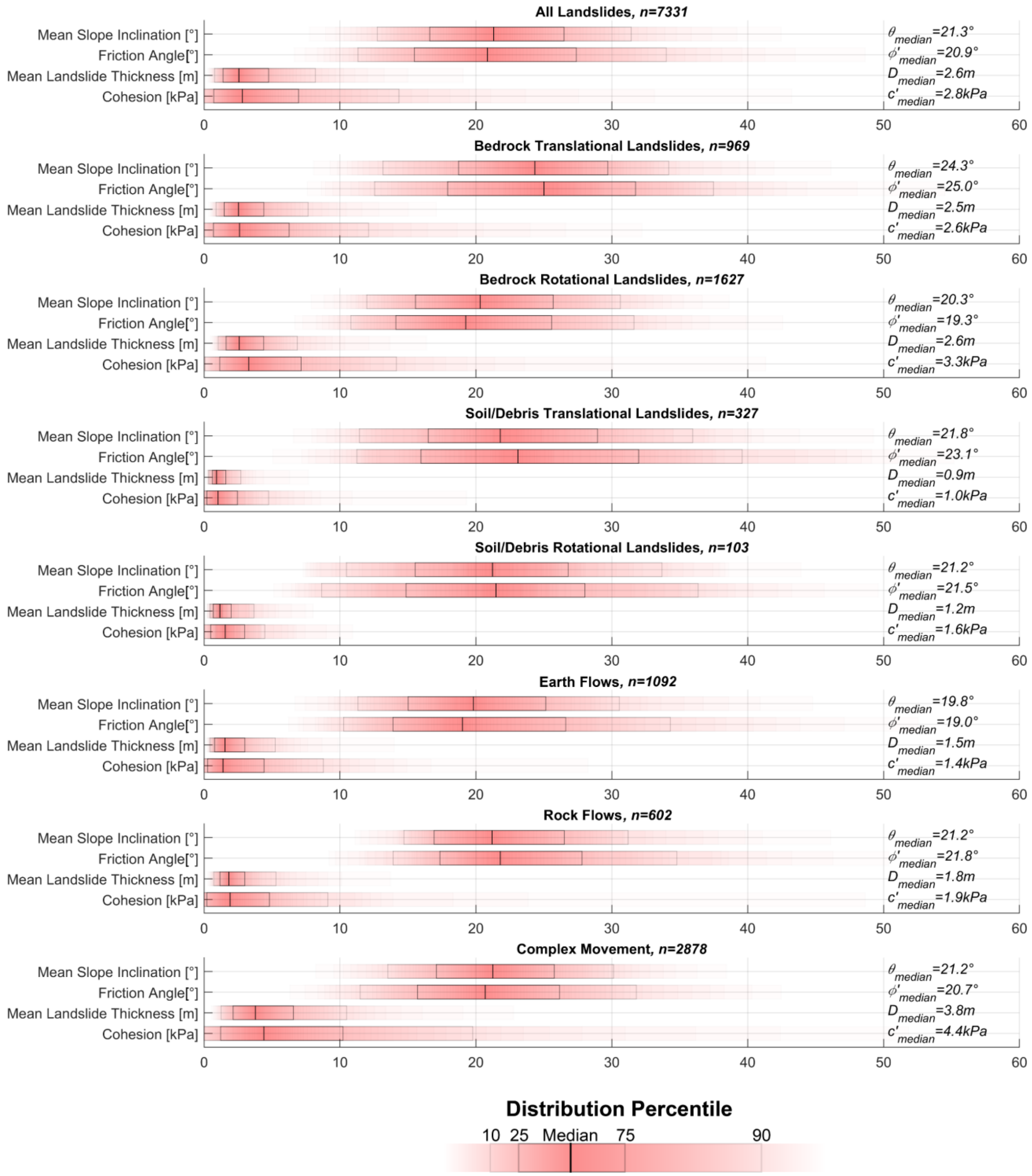

Figure SI.9. Distributions of strength (effective friction angle, ϕ', and cohesion, c') and landslide morphology (mean thickness, D, and mean surface inclination, θ) for n landslide samples by Varnes landslide classification (DOGAMI 2009). Bedrock landslides and particularly landslide complexes demonstrate higher mean thickness and cohesion than their soil/debris counterparts. Modest differences are shown between median rock and soil/debris landslide friction angles, but generally the higher percentiles of soil/debris are higher than bedrock counterparts.

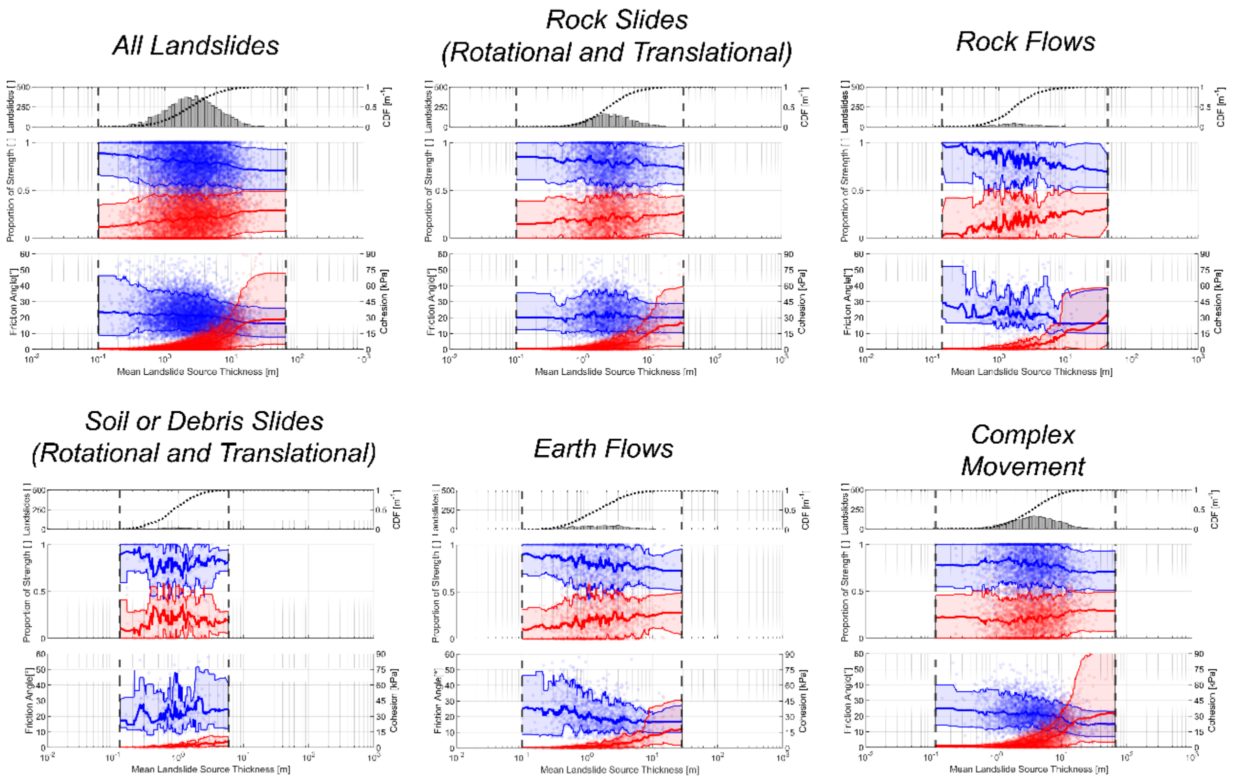

Figure SI.10. Relationships between mean landslide thickness and strength for different subjective landslide classifications. Most subjective mechanism classifications demonstrate an exchange between frictional and cohesive strength with mean thickness, although the trends are noisy for the few samples for soil/debris slides. Most mechanisms demonstrate a decrease in friction angle with landslide thickness and a commensurate increase in cohesion. Complex movements tend to have mean thicknesses associated with bedrock (i.e. >1 m) and a pronounced decrease in friction angle with thickness.

Table SI.2a. Wilcoxon rank sum test comparing friction angle distributions of different landslide classifications and their level of statistical significance of their differences. A (-) reflects no statistical difference under a 5% significance level, (*) represents statistical differences under a 5% significance level, (**) represents statistical differences under a 1% significance level, and (***) represents statistical differences under a 0.1% significance level.

FRICTION ANGLE								
	**All Landslides**	**Bedrock Translational Landslides**	**Bedrock Rotational Landslides**	**Soil/Debris Translational Landslides**	**Soil/Debris Rotational Landslides**	**Earth Flows**	**Rock Flows**	**Complex Movement**
**All Landslides**	1	3.01×10-24 (***)	9.16×10-08 (***)	2.91×10-02 (*)	7.03×10-01 (-)	1.39×10-09 (***)	1.02×10-02 (*)	2.54×10-01 (-)
**Bedrock Translational Landslides**	-	1	1.03×10-32 (***)	4.63×10-03 (**)	2.77×10-04 (***)	2.66×10-30 (***)	7.40×10-07 (***)	1.29×10-25 (***)
**Bedrock Rotational Landslides**	-	-	1	3.76×10-05 (***)	3.41×10-01 (-)	1.07×10-01 (-)	3.87×10-08 (***)	4.45×10-05 (***)
**Soil/Debris Translational Landslides**	-	-	-	1	1.52×10-01 (-)	4.56×10-06 (***)	7.21×10-01 (-)	1.01×10-02 (*)
**Soil/Debris Rotational Landslides**	-	-	-	-	1	1.50×10-01 (-)	2.13×10-01 (-)	9.24×10-01 (-)
**Earth Flows**	-	-	-	-	-	1	3.62×10-10 (***)	2.06×10-07 (***)
**Rock Flows**	-	-	-	-	-	-	1	2.43×10-03 (**)
**Complex Movement**	-	-	-	-	-	-	-	1

Table SI.2b. Wilcoxon rank sum test comparing cohesion distributions of different landslide classifications and their level of statistical significance of their differences. A (-) reflects no statistical difference under a 5% significance level, (*) represents statistical differences under a 5% significance level, (**) represents statistical differences under a 1% significance level, and (***) represents statistical differences under a 0.1% significance level.

COHESION								
	**All Landslides**	**Bedrock Translational Landslides**	**Bedrock Rotational Landslides**	**Soil/Debris Translational Landslides**	**Soil/Debris Rotational Landslides**	**Earth Flows**	**Rock Flows**	**Complex Movement**
**All Landslides**	1	1.08×10-02 (*)	5.16×10-01 (-)	4.92×10-31 (***)	1.17×10-08 (***)	7.30×10-27 (***)	6.11×10-06 (***)	1.68×10-39 (***)
**Bedrock Translational Landslides**	-	1	7.09×10-03 (**)	1.06×10-20 (***)	1.92×10-06 (***)	3.79×10-10 (***)	3.09×10-02 (*)	7.01×10-25 (***)
**Bedrock Rotational Landslides**	-	-	1	2.72×10-31 (***)	1.37×10-09 (***)	1.01×10-22 (***)	4.43×10-06 (***)	1.16×10-20 (***)
**Soil/Debris Translational Landslides**	-	-	-	1	8.63×10-02 (-)	1.03×10-06 (***)	6.96×10-13 (***)	8.77×10-54 (***)
**Soil/Debris Rotational Landslides**	-	-	-	-	1	9.83×10-02 (-)	3.29×10-04 (***)	2.19×10-17 (***)
**Earth Flows**	-	-	-	-	-	1	1.52×10-03 (**)	6.86×10-68 (***)
**Rock Flows**	-	-	-	-	-	-	1	2.61×10-26 (***)
**Complex Movement**	-	-	-	-	-	-	-	1

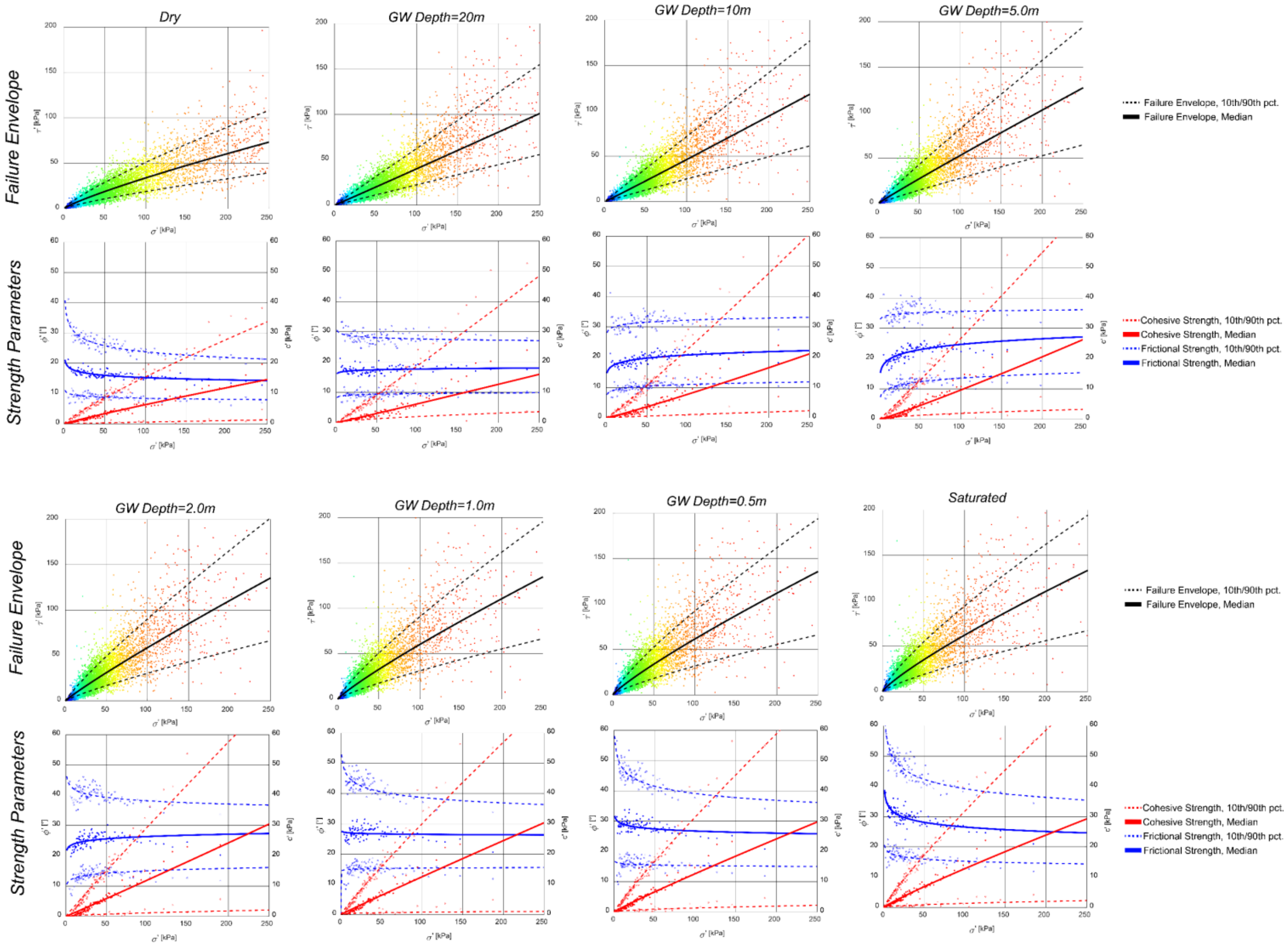

Figure SI.11. Failure envelopes (top row) and strength properties (bottom row) as related to mean effective normal stress (σ') for a suite of groundwater depths. A nonlinear failure envelope is shown for most potential groundwater conditions (the exception being groundwater depths of 10m and 20m). In some cases, relationships between friction angle are relatively insensitive to normal effective stress (e.g. groundwater depths of 1 m to 20 m), but nonlinearity is maintained as a function of cohesion.

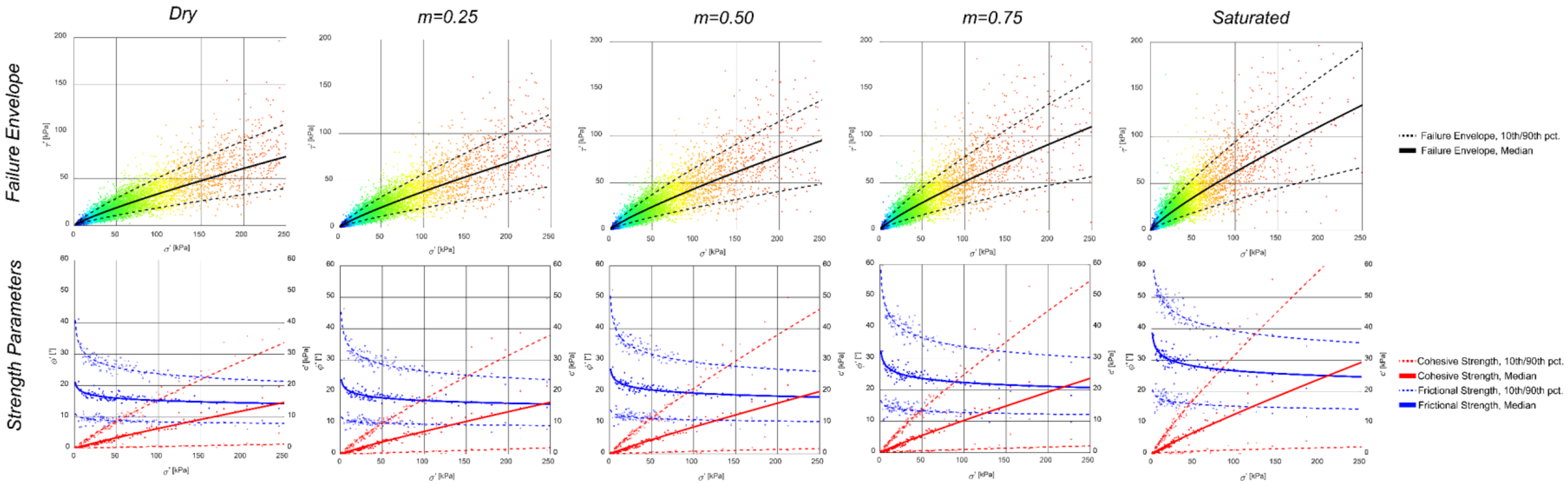

Figure SI.12. Failure envelopes (top row) and strength properties (bottom row) as related to mean effective normal stress (σ') for a suite of saturation ratios (m). A nonlinear failure envelope is shown for all percentiles and assumed groundwater conditions, reflecting diminishing frictional resistance with mean effective normal stress and consequently, landslide thickness.

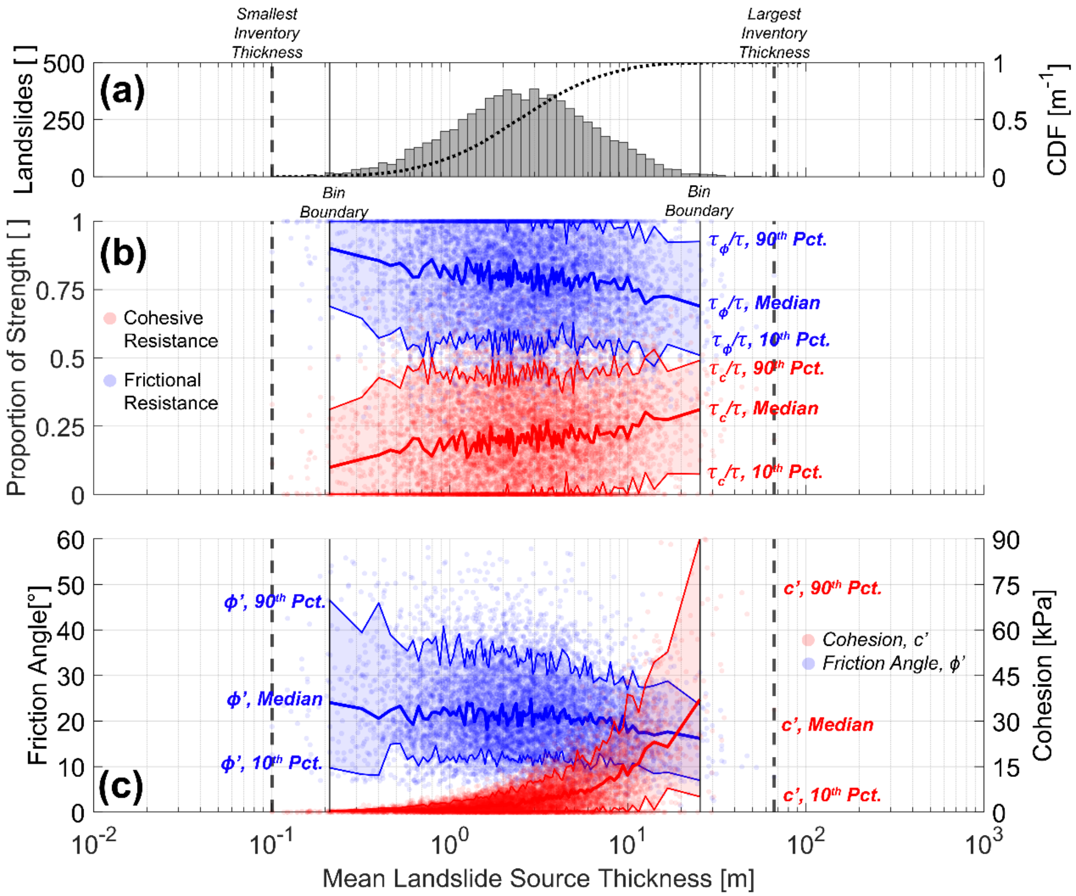

Figure SI.13. Exchange in strength with landslide size considering bins of landslide mean thickness. (a) Histogram and cumulative distribution function (CDF) of landslides by mean thickness. Approximately 25% of landslides have a mean thickness of less than 1m (predominantly soil), while 70% have mean thicknesses of 1 to 10 m (predominantly saprolite, weathered bedrock and potentially fresh bedrock). (b) Proportion of total shear strength (unitless, 0 to 1) attributed to cohesion and friction in comparison to landslide thickness. The shaded areas represent the bounds between 10th and 90th moving percentiles (1% bins of all data) of cohesive and frictional resistance, respectively. A gradual transfer from frictional resistance to cohesive resistance is observed with increasing landslide thickness, the largest exchange occurring within weathered bedrock suggestive of a depth of potentially aggressive weathering and strength change. (c) Exchange in friction angle and cohesion with mean landslide thickness.

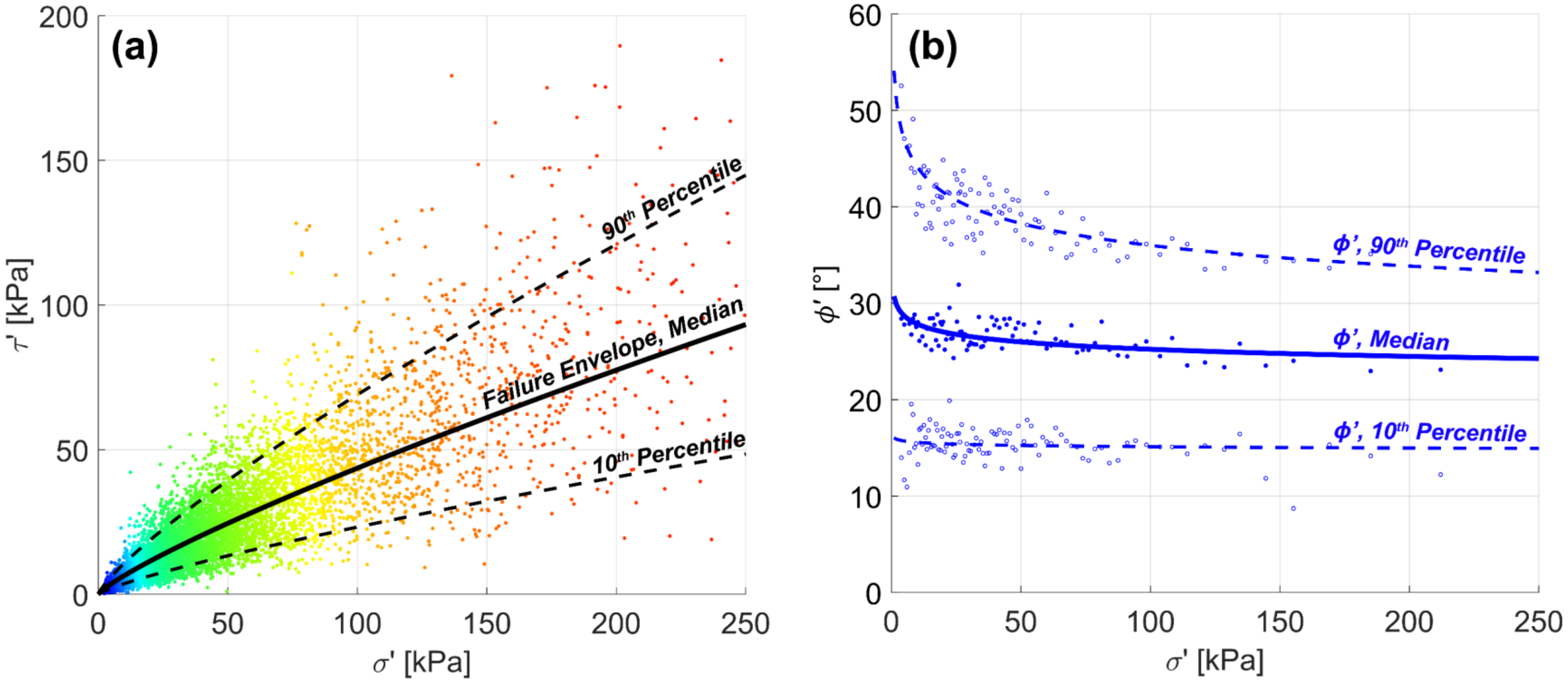

Figure SI.14. (a) Fitted failure envelopes for 10th, 50th and 90th percentiles based on bins of 1% of all effective normal stress data for friction angles determined only from the landslide source area (i.e. no back-analysis of cohesion) for m=0.5 conditions. The failure envelope is still nonlinear, reflecting the importance of effective normal stress (a function of groundwater, inclination, and mean landslide thickness) on governing shear strength. The nonlinear envelope is similar to those proposed for rock (i.e. Barton 2006). **(b)** The nonlinearity for this specific set of assumptions and conditions stems from a nonlinear decrease in friction angle with effective normal stress, as shown for 1% bins of all effective normal stress data.

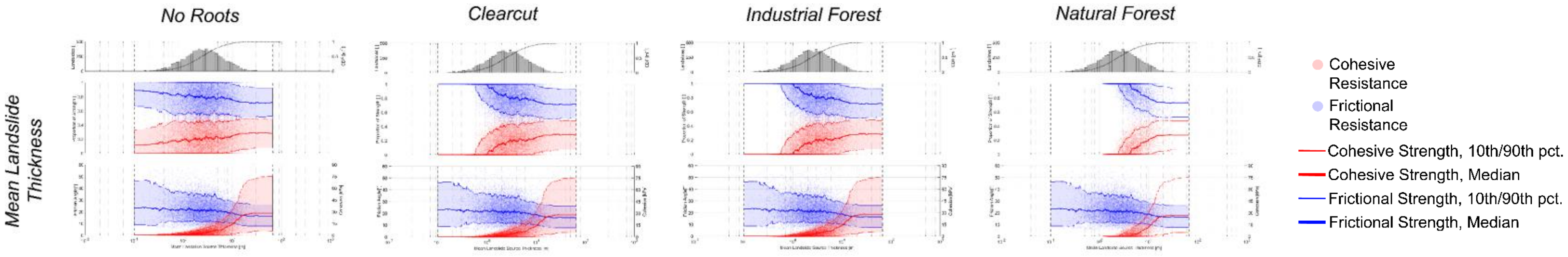

Figure SI.15. Relationships between mean landslide thickness and strength for different levels of lateral root cohesion (c_r) representative of different forest management conditions in the Oregon Coast Range (Roering et al. 2003). For management conditions of *No Roots*, *Clearcut*, *Industrial Forest*, and *Natural Forest*, root cohesions were 0, 3.35, 7.66, and 54.89 kPa, respectively. Lateral root cohesion was only applied to the area of the rupture surface with depths of 0.5 m or less, consistent with the general shallow rooting depths described by Roering et al. (2003). These root cohesion values have been conjectured to possible overestimate the mechanical reinforcement of roots (by as much as 75%) owing to their progressive breakage and pullout (e.g. Pollen et al. 2005, Cohen et al. 2011, Giarossich et al. 2019), thus reflect an upper bound for the proposed management conditions. As shown, stronger root cohesions amplify the proposed exchange between frictional and cohesive resistance with landslide thickness as much of the back-analyzed mineral cohesion is now supported by lateral root cohesion.

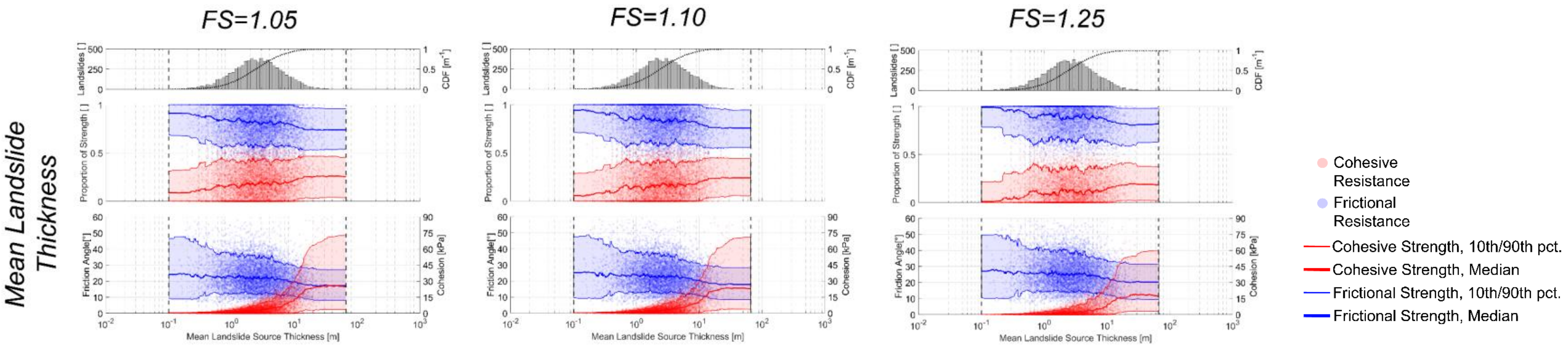

Figure SI.16. Relationships between mean landslide thickness and strength for different factors of safety (*FS*) applied to the friction angle of landslide deposits. This sensitivity analysis tests the possibility that landslide deposits are stable and consequently not in a state of limiting equilibrium. As shown, increased *FS* applied to deposit friction angles yields lower cohesion values and offsets the exchange between cohesive and frictional resistance, but an exchange is still present.

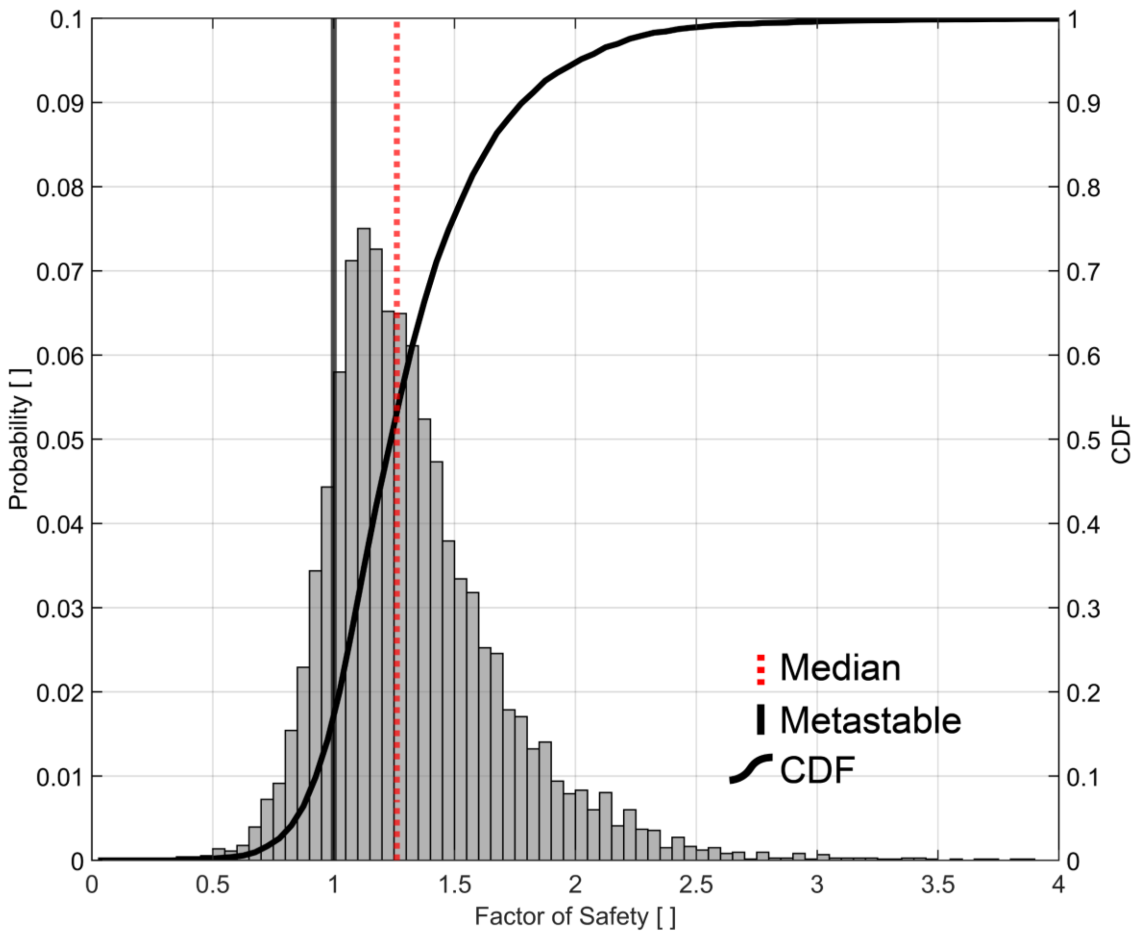

Figure SI.17. Distribution of factor of safety (*FS, unitless*) probability (unitless)considering no cohesion at the source area at the time of failure, simplified under purely frictional conditions as $FS = tan(\phi'_{source})/tan(\phi'_{deposit})$. This distribution shows that the median *FS* for deposits would be ≈1.26, which is on par with the stability of engineered slopes (Allen 2015). As landslide deposits are often prone to continued failure from being placed in a precarious state (Temme et al. 2020), such a high *FS* is possible, but perhaps not always likely. This suggests that back-analysis of friction from deposits and subsequent use for back-

analysis of cohesion from source areas, while an assumption, may serve as a reasonable technique to glean first order estimates of unique cohesion and friction pairs for landslide inventories.

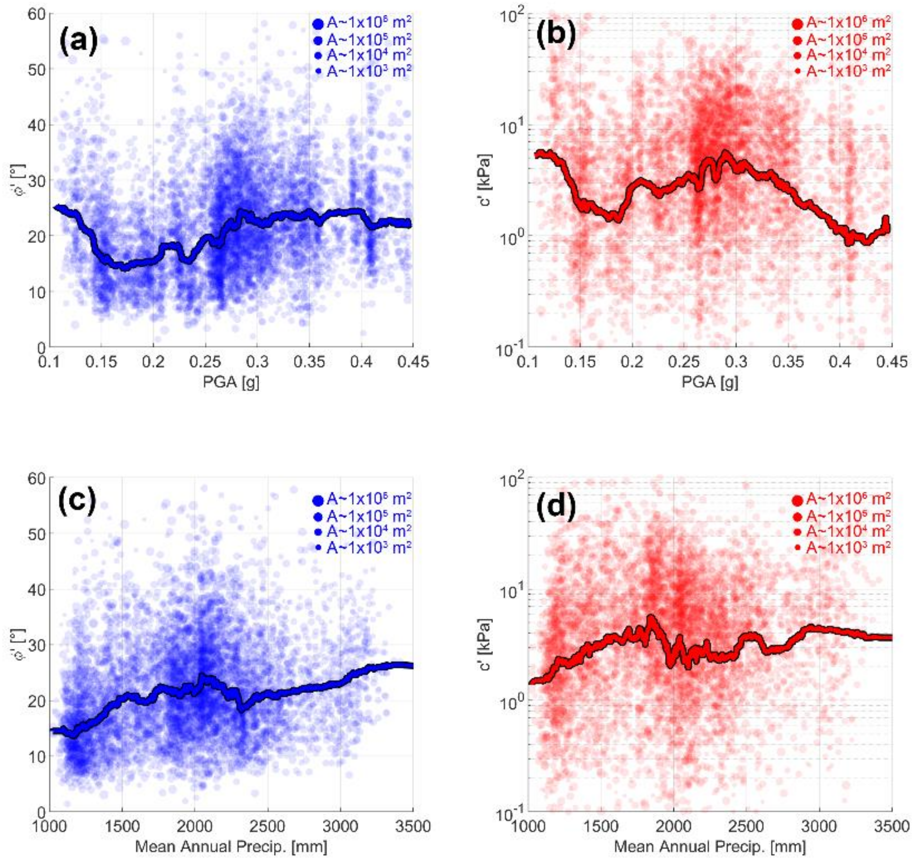

Figure SI.18. Strength relationships versus mean annual precipitation and modeled peak ground acceleration (PGA) for a M_w 9.0 earthquake. As shown there are no clear trends between PGA and friction or cohesion, suggesting that there is no obvious control of coseismic triggering in the landslide inventories. A modest trend between mean annual rainfall and strength does persist (i.e. higher strength for more rainfall).

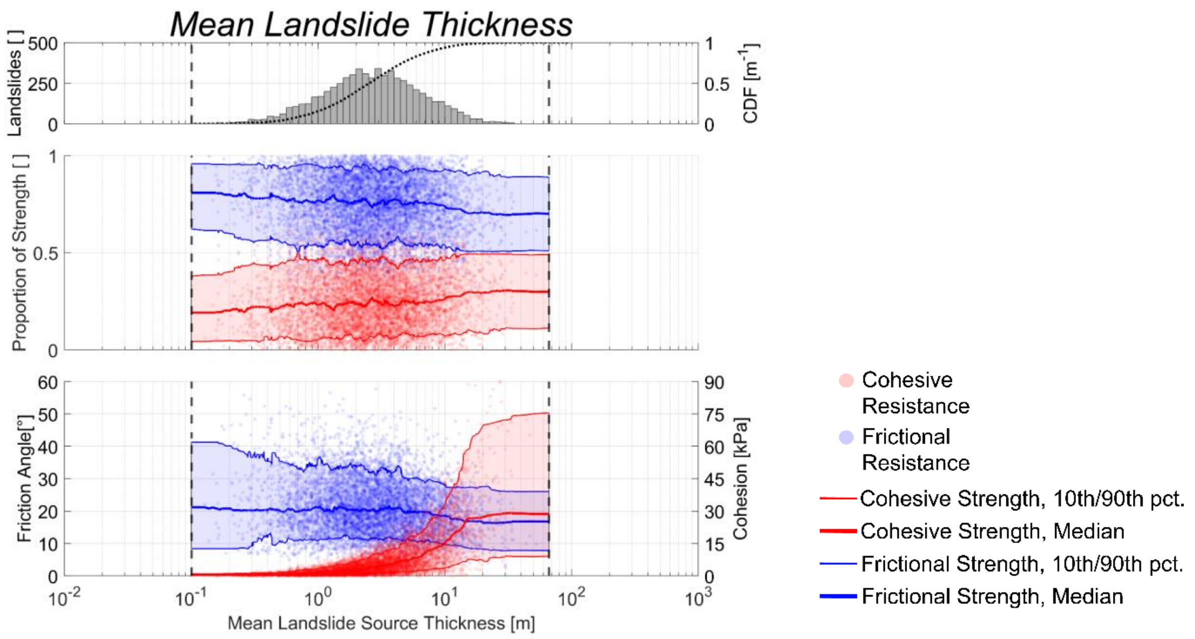

Figure SI.19. Relationships between mean landslide thickness and strength (upper panel) and landslide area and strength (lower panel) excluding all landslides where the friction angle for existing topography was smaller than that from reconstructed terrain (≈14% of all landslides). As shown, similar trends to those presented still persist, although the exchange of proportional strength with mean landslide thickness is slightly more gradual.

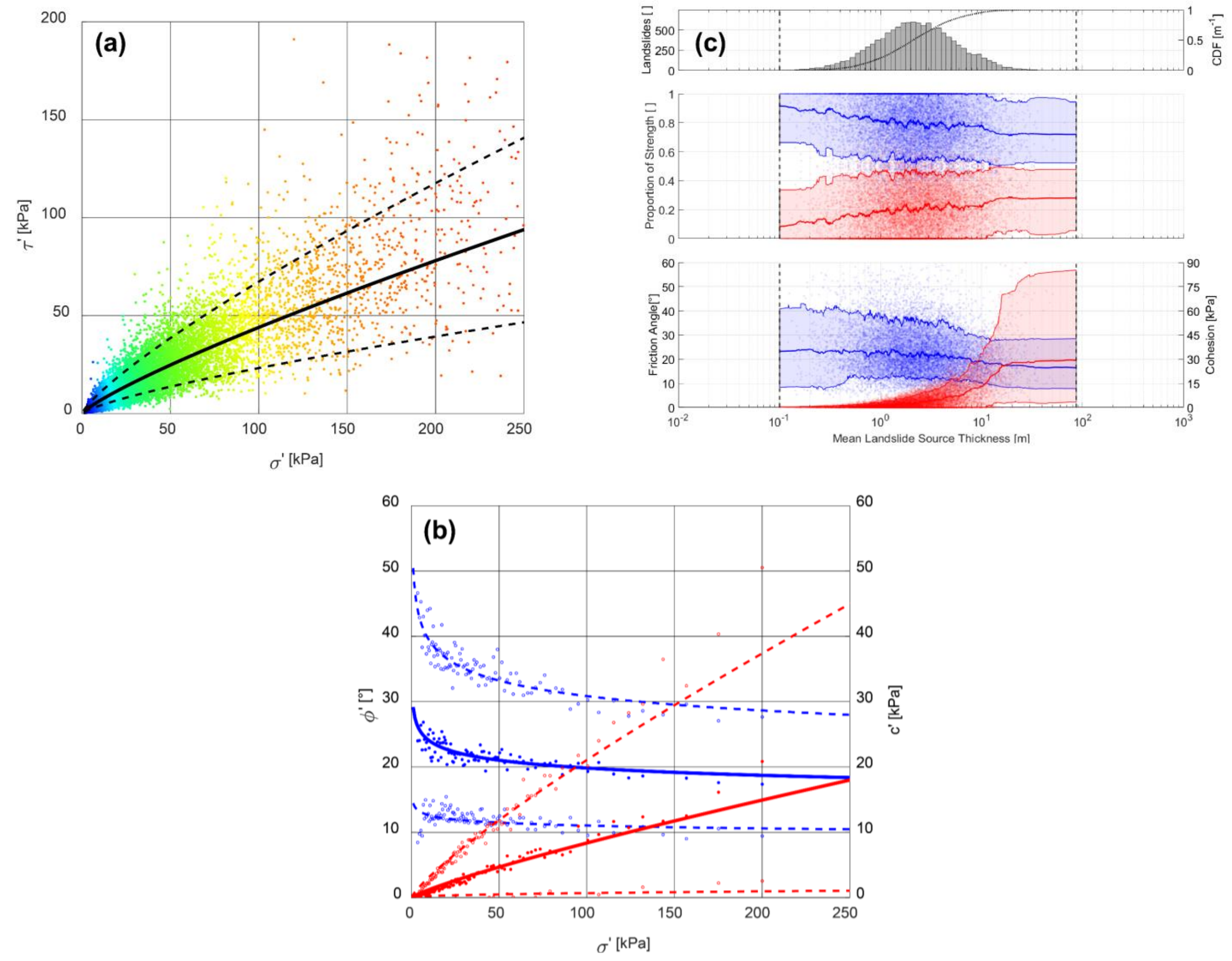

Figure SI.20. (a) Failure envelope, (b) strength versus effective normal stress, (c) strength relationships with mean landslide thickness for all landslides without exclusion based on bulking (0.5-5.5) or overlap of source and deposit areas (0.25-1). The observed nonlinearity of the failure envelope and trends between strength and normal stress or landslide thickness are similar to those presented in the paper, suggestive of limited sampling bias.

REFERENCES

Allen, T. (2015). Geotechnical design manual. *Washington Department of Transportation, M*, 46-03.

Barton, N. (2006). *Rock quality, seismic velocity, attenuation and anisotropy*. CRC press.

Cohen, D., Schwarz, M., & Or, D. (2011). An analytical fiber bundle model for pullout mechanics of root bundles. *Journal of Geophysical Research: Earth Surface*, *116*(F3).

Franczyk, Jon J, William J Burns, and and C Nancy Calhoun. 2019. Statewide Landslide Information Database for Oregon (SLIDO), Release 4.0 Oregon Department of Geology and Mineral Industries SLIDO Digital Data Series.

Giadrossich, F., Cohen, D., Schwarz, M., Ganga, A., Marrosu, R., Pirastru, M., & Capra, G. F. (2019). Large roots dominate the contribution of trees to slope stability. *Earth Surface Processes and Landforms*, *44*(8), 1602-1609.

Madin, I., Burns, W. J., & McConnell, V. S. (2013). Ground motion, ground deformation, tsunami inundation, coseismic subsidence, and damage potential maps for the 2013 Oregon Resilience Plan for Cascadia Subduction Zone Earthquakes. Oregon Department of Geology and Mineral Industries.

Pollen, N., & Simon, A. (2005). Estimating the mechanical effects of riparian vegetation on stream bank stability using a fiber bundle model. *Water Resources Research*, *41*(7).

Roering, J. J., Schmidt, K. M., Stock, J. D., Dietrich, W. E., & Montgomery, D. R. (2003). Shallow landsliding, root reinforcement, and the spatial distribution of trees in the Oregon Coast Range. *Canadian Geotechnical Journal*, *40*(2), 237-253.
